# Hospital Services to Improve Nutritional Intake and Reduce Food Waste: A Systematic Review

**DOI:** 10.3390/nu15020310

**Published:** 2023-01-07

**Authors:** Emanuele Rinninella, Pauline Raoul, Valeria Maccauro, Marco Cintoni, Andrea Cambieri, Alberto Fiore, Maurizio Zega, Antonio Gasbarrini, Maria Cristina Mele

**Affiliations:** 1 Dipartimento di Medicina e Chirurgia Traslazionale, Università Cattolica del Sacro Cuore, Largo F. Vito 1, 00168 Rome, Italy; 2 UOC di Nutrizione Clinica, Dipartimento di Scienze Mediche e Chirurgiche, Fondazione Policlinico Universitario A. Gemelli IRCCS, 00168 Rome, Italy; 3 Scuola di Specializzazione in Medicina Interna, Università Cattolica del Sacro Cuore, Largo F. Vito 1, 00168 Rome, Italy; 4 Direzione Sanitaria, Fondazione Policlinico Universitario A. Gemelli IRCCS, 00168 Rome, Italy; 5 UOC Qualità e Accreditamento, Fondazione Policlinico Universitario A. Gemelli IRCCS, 00168 Rome, Italy; 6 UOC Servizio Infermieristico, Tecnico, Riabilitativo, Aziendale (S.I.T.R.A.), Fondazione Policlinico Universitario A. Gemelli IRCCS, 00168 Rome, Italy; 7 UOC di Medicina Interna e Gastroenterologia, Dipartimento di Scienze Mediche e Chirurgiche, Fondazione Policlinico Universitario A. Gemelli IRCCS, 00168 Rome, Italy

**Keywords:** food waste, nutritional intake, hospital food strategies, personalised nutrition, malnutrition, diet, room service, nutritional counseling, dietitian

## Abstract

Background and Aims: Patients’ nutritional intake is a crucial issue in modern hospitals, where the high prevalence of disease-related malnutrition may worsen clinical outcomes. On the other hand, food waste raises concerns in terms of sustainability and environmental burden. We conducted a systematic review to ascertain which hospital services could overcome both issues. Methods: A systematic literature search following PRISMA guidelines was conducted across MEDLINE, Web of Science, and Scopus for randomised controlled trials (RCTs) and observational studies comparing the effect of hospital strategies on energy intake, protein intake, and plate/food waste. The quality of included studies was assessed using the Newcastle-Ottawa Scale for cohort studies and the Cochrane Risk of Bias tool from the Cochrane Handbook for Systematic Reviews of Interventions for RCTs. Results: Nineteen studies were included, assessing as many hospital strategies such as food service systems—including catering and room service—(*n* = 9), protected mealtimes and volunteer feeding assistance (*n* = 4), food presentation strategies (*n* = 3), nutritional counseling and education (*n* = 2), plant-based proteins meal (*n* = 1). Given the heterogeneity of the included studies, the results were narratively analysed. Conclusions: Although the results should be confirmed by prospective and large sample-size studies, the personalisation of the meal and efficient room service may improve nutritional intake while decreasing food waste. Clinical nutritionist staff—especially dietitians—may increase food intake reducing food waste through active monitoring of the patients’ nutritional needs.

## 1. Introduction

Approximately 40% of inpatients are already malnourished at hospital admission [1]. Hospital malnutrition is a neglected and prevalent problem influencing not only patients’ clinical outcomes (length of stay, morbidity, mortality, and quality of life) but also the sustainability of healthcare as a whole [2]. In the United Kingdom (UK), the expenditure related to malnutrition has been evaluated as 15% of the total health and social expenditure [3]. In Western countries, hospital malnutrition is the visible expression of disease-related malnutrition, often due to the inflammatory burden of diseases or the inability to intake (or uptake) nutrients from a standard diet [4]. In such conditions, specific and nutritional support is needed to counteract the onset and the progression of malnutrition. For this purpose, hospital meals should be part of therapy and every effort should be made to give patients fresh, palatable, and nutritive meals. However, nutritional intake in hospital is often undervalued and patients fail to reach their energy and protein requirements for many reasons, among which are loss of appetite, prescribed fasting, dislike of food, lack of support in feeding, nausea, etc. [5]. Moreover, collective catering services in healthcare facilities face various difficulties (dietary and nutritional needs, quality of meals, hygiene-sanitary standards, etc.). Thus, the nutritional status worsens during hospital stay and this worsening is more pronounced in malnourished patients, creating a vicious circle [6]. On the other hand, uneaten food has serious implications in terms of sustainability and environmental issues. Food waste in the food services industry has been called an “unsustainability hotspot” [7]; it has been calculated that the amount of food waste produced in one hospital that serves 6640 patient meals per week can equal more than 48,000 lbs (24 tons) [8]; food wastage increases the amount of food grown or raised, the fuel in the transport of food to hospitals, and the amount of methane and carbon dioxide from landfilling the uneaten food [9]. Healthcare facilities record the highest volumes of food waste compared with other types of collective catering, with food wastage ranging between 17% and 67% depending on the service system [10]. The critical concerns of this issue include also climate change [11], food security [12], monetary losses [13] and the overall economic impact of such waste [14]. 

Even though theoretical awareness by the scientific community in this matter is rising, there are no homogeneous indications to improve nutritional intake and reduce hospital food waste in the healthcare facilities, and each hospital has proper food service or catering, often based on merely economic evaluations. The aim of this systematic review is to collect scientific evidence about the services until now offered by hospitals to ameliorate patients’ nutrient intake and reduce food waste. 

## 2. Methods

This systematic review followed the Preferred Reporting Items for Systematic Reviews and Meta-Analyses (PRISMA) guidelines [15]. This systematic review was registered at PROSPERO as CRD42022376185.

### 2.1. Inclusion and Exclusion Criteria 

The inclusion criteria are presented according to the PICOS criteria (Table 1). 

Exclusion criteria were studies including patients living in residential home, patients fed with artificial nutrition, reviews, comments, editorials, case series, or meeting abstracts.

### 2.2. Data Sources and Search Strategy 

The search was carried out using three electronic databases, MEDLINE (via PubMed), ISI Web of Science, and Scopus. Multiple search terms were used, including hospital food, meal service, hospital meals, hospital catering, food waste, protein intake, and energy intake. The search string for each database is described in Table S1. Hand searching of eligible studies was carried out to find studies that may not have been found in the databases. 

### 2.3. Study Selection, Data Extraction and Reporting

All articles were retrieved from an Excel sheet for screening of titles and abstracts for eligibility based on inclusion criteria. Duplicates were removed. The first screening of studies was independently carried out by two reviewers by reading titles and abstracts. All titles or abstracts assessed as ineligible were excluded. Differences in judgment during the selection process between the two reviewers were settled by discussion and consensus. The full text of each selected article was retrieved, and any ineligible articles were excluded based on exclusion criteria indicated above. After full-text analysis, the following information was extracted from the included articles: title, first author of publication, year of publication, country, study design, sample size, patient type, study setting, intervention type, time assessment, endpoints methods of assessment, and results of each study. Data were reported using an Excel© (Microsoft Office, Redmond, WA, USA) spreadsheet specifically developed for this study. 

### 2.4. Quality Assessment 

The quality of included studies was assessed by two reviewers. Any discrepancy was resolved by discussion. The Newcastle-Ottawa Scale (NOS) for cohort studies [16] and its version adapted for cross-sectional studies [17] assesses the quality and risk of bias of observational studies. A ‘star system’ has been developed in which a study is judged on three domains: the selection of the study groups; the comparability of the groups; and the ascertainment of either the exposure or outcome of interest for case-control or cohort studies, respectively. The quality assessment of the randomised controlled trials was assessed according to the Cochrane risk of bias tool from *Cochrane Handbook for Systematic Reviews of Interventions* [18]. 

## 3. Results 

### 3.1. Study Selection 

**Figure 1:**
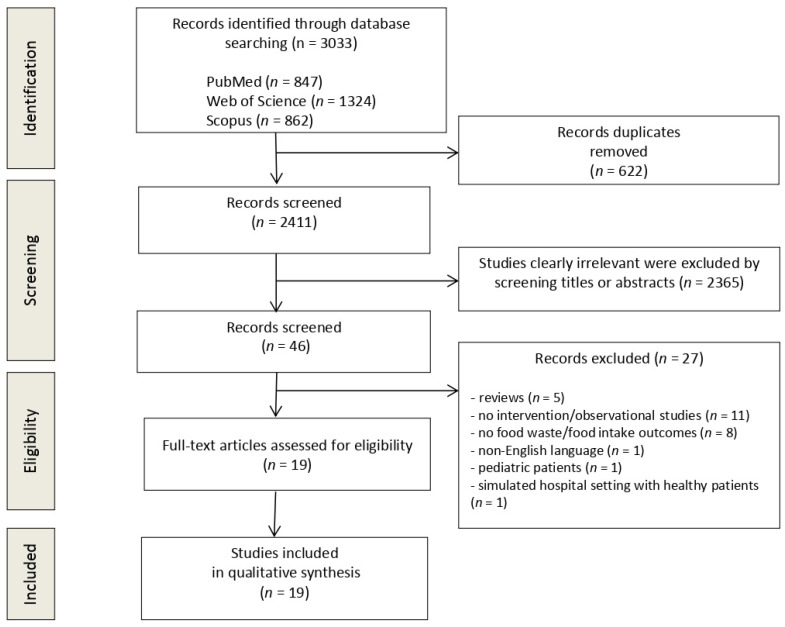
Preferred reporting items for systematic reviews and meta-analyses (PRISMA) flow diagram.

The flow diagram in Figure 1 displays the results of the literature search and study selection process. A total of 3033 studies were initially identified. After duplicate removal, 2411 studies remained for title and abstract screening. Twenty-seven studies were excluded for the following reasons: reviews (*n* = 5), no intervention (*n* = 11), no food waste/food intake outcomes (*n* = 8), non-English language (*n* = 1), paediatric patients (*n* = 1), simulated hospital setting with healthy participants (*n* = 1). Nineteen studies were identified for inclusion in the systematic review. 

### 3.2. Study Characteristics 

Table 2 detailed the characteristics of studies.

### 3.3. Quality Assessment 

The NOS score of each study is detailed in Table S2 in Supplementary Materials. The quality score was good for all studies except for one study [29]. A NOS score of nine stars was assigned to six out of 17 included studies [19,23,26,27,32,36]. Although the selection of the non-exposed cohort was good for all studies, the sample size was reduced for five studies [20,21,24,28,29]. Farrer et al. experienced a high withdrawal rate due to patients not meeting the inclusion criteria [29]. Two studies used a pre-post study design [25,31]. Four studies have confounders to consider for the comparability of exposed and non-exposed cohorts (*p >* 0.05) in terms of age [29,33,34,35], medical classification [33,34], gender [29], and reasons for special diet [29]. Concerning the assessment of outcomes, the studies estimated the energy requirements without using indirect calorimetry. Moreover, Young et al. assessed the food intake of patients for only one day of hospital admission [25]. Farrer et al. 2016 reported responses mostly collected verbally from patients or their relatives by the investigators [29]. Moreover, the follow up of cohorts was considered adequate except for two studies [31,33].

Both included RCTs [22,30] and were assessed according to the Cochrane risk of bias tool from *Cochrane Handbook for Systematic reviews of Interventions* [18]. The risk of bias is detailed in Table S3 Supplementary Data. Both studies have an adequate random sequence generation and allocation concealment (selection bias) and an adequate reporting of results. Blinding of participants was the highest risk of bias for the two studies since as the nature of the intervention was nutritional, it was not possible to blind participants and personnel. Regarding other bias, the sample size calculation was not performed in the study of Rufenacht et al. [22], reducing the validity of their results.

### 3.4. Results

#### 3.4.1. Food Service Systems including Catering and Room Service (*n* = 9) 

An observational study conducted in the UK [19] concluded that a bulk trolley system (in which patients were able to freely choose food and its amount from the trolley), was effective in reducing plate food waste compared to a plated meal system (in which meals were ordered in advance 24 h before consumption) (5.9% vs. 11.6%). However, the waste left on the trolley remained high (20.5%) with the trolley service; moreover, the nutrient intakes were similar in the two groups, with both below that recommended by dietary reference values. Later in the UK, a peculiar catering system, called “*Steamplicity*” was developed. *Steamplicity* uses raw, semi- or fully cooked chilled foods, plated in a central production unit and delivered to the hospital ward into a sealed pack (plate) incorporating a valve. The food remains chilled for up to four days and then heated in a microwave when required. Two observational studies [20,21] compared *Steamplicity* vs. a traditional cook-chill food. The first [20] reported a higher food intake (daily mean of 282 g vs. 202 g at lunch; 310 g vs. 226 g at dinner) and a lower food wastage (33% vs. 49%) in *Steamplicity* system than in cook-chill service; the second [21] did not confirm these results, reporting a lower energy intake in *Steamplicity* than in bulk cook-chill system (*p* = 0.04) and no difference in protein intake between the two systems. However, as the authors noted, the energetic values of the two methods were different: energy provided by bulk service dessert was higher than that of *Steamplicity.* Of note, in this second study, the observational period was only one meal/patient (at lunch), whereas in the first study [20], the time of observation was three consecutive days in two-week periods for each arm.

A pilot trial [28] assessed the impact on patients’ outcomes and the cost of a modified hospital menu with higher energy foods (i.e., pikelets, omelettes, muffins, and cake) replacing less energy-dense foods (tea, coffee, sea salads), compared to a standard cook-chill menu. In the intervention group, oral nutritional supplements (ONS) were also added and patients could choose food items by a visual menu. Authors found a higher mean intake of energy (132 vs. 105 Kj/kg/day; *p* = 0.003) and protein intake (1.4 vs. 1.1 g protein/kg/day; *p* = 0.035) in the intervention vs. control group. Of note, the additional cost of the intervention was estimated at £4.15 /participant/day. 

Two Australian retrospective studies evaluated the effectiveness of a room service in a case mix of hospital wards [33,35]. Room service is a foodservice model in which patients order meals from an “a la carte menu”, and consume them within 45 min of ordering. McGray et al. compared room service vs. a traditional foodservice model (meals ordered by a 14-day cycle menu 24 h before) over a period of four days (pre- and post-intervention); they found a higher mean energy intake (1588 kcal/d vs. 1306 kcal/d; *p* < 0.005) and higher mean protein intake (65.9 g/d vs. 52.3 g/d; *p* < 0.003) in the room service period. Moreover, in the intervention period, there was lower total mean plate waste (12% vs. 29%; *p* < 0.001) and meal costs decreased by 15% [33]. Three years later, Neaves confirmed these results in a period of observation of five weekdays, comparing room service with a thaw-retherm service control group. In the intervention group, a higher average energy and protein intake was reached (*p* < 0.001). Additionally, a reduced plate waste (15% vs. 40%) and production waste (5.6% vs. 15%, *p* < 0.001) were found, resulting in reduced total average production waste (*p* < 0.001), with meal costs decreased by 9% for room service [35]

Three studies evaluated the effect of an electronic bedside spoken meal ordering system (BMOS) compared to a paper menu on food intake and food waste in hospitalised patients [26,32,34]. All three studies found a significant increase in energy intake and protein intake in the BMOS group compared with the paper menu group (*p* < 0.05) [26,32,34]. Moreover, Mc Cray et al. assessed a significant increase in percentage of energy requirements (64% vs. 78%, *p* = 0.002) and protein requirements (70% vs. 99%, *p* < 0.001) met in the BMOS group compared with the paper menu group [34]. However, the effect of BMOS intervention on food waste was discussed. Indeed, Mc Cray et al. found a significant decrease in total average plate waste (30% vs. 17%, *p* < 0.001) [34], while Barrington et al. assessed no significant differences in average plate waste between both groups (35.3% vs. 34.3%, *p* = 0.75) [32].

#### 3.4.2. Protected Mealtimes and Volunteer Feeding Assistance (*n* = 4)

Three studies investigated whether protected mealtimes (PM) could increase food intake in adult/elderly patients at risk of malnutrition [23,24,30], one study in the UK [23] and two studies in Australia [24,30]. PM are defined as “periods in hospital ward when all non-urgent clinical activity stops. During these times, patients can eat without being interrupted and staff can offer assistance” [23]. These interventions would rather modify the patients’ environment than the food choice or the meal presentation. All three studies found that PM do not increase energy and protein intake compared to a control period (standard food service and no feeding assistance). Surprisingly, the study of Hickson [23] found a decrease in protein intake at the lunchtime meal in PM period (14.0 vs. 7.5 g, *p* = 0.04), without a clear reason. On the other hand, a volunteer feeding assistance could improve energy and protein intakes of elderly inpatients, as demonstrated by Manning et al. [24] in an Australian monocentric observational study comparing the feeding assistance offered by volunteers (intervention) and that provided by nurses (control). One of the main reasons for these results may be the greater average time spent at lunchtime with each patient by volunteers (12.3 min versus 4.7 min of nurses). 

#### 3.4.3. Food Presentation (*n* = 3)

Three included studies measured the effect of meal presentation/appearance on plate waste [27,29,36]. Razzali et al. investigated the effect of three diet textures (blended diet, mixed porridge, and minced diet) on the percentage of plate waste among 95 hospitalised patients prescribed with texture-modified diet [36]. Based on weighing method, higher plate waste (65%) was observed in the blended diet (65%) than the minced diet (56%) and mixed porridge (35%). Higher protein waste (61.1%) was measured in the minced diet compared with the other diets (based on weighing method). Another study [29] assessed the food waste in institutionalised patients with a texture modified diet, especially with smooth pureed meals. They found a significant increase in food intake from 1/4 meal eaten to > 3/4 meal by patients fed with smooth puree meals in the mould form (week 2) than unmoulded form (week 1) (*p* = 0.03) [29]. At week 2, they also showed a decrease in 126 g food waste in patients with meals in the moulded form (*n* = 27) compared with patients with unmoulded meals (*n* = 37), although this result was not significant (*p* > 0.05). 

Navarro et al. [27] showed that an improved presentation of lunch (by advice received by Paul Bocuse) significantly increased 19% of food intake compared with standard presentation (*p* < 0.05) and significantly decreased starch and main course waste (*p* < 0.05) in 206 Israelian hospitalised patients. 

#### 3.4.4. Nutritional Counselling and Education (*n* = 2)

Two studies assessed the effect of the implementation of awareness interventions of nutritional support on food intake [22,31]. A RCT of 36 hospitalised malnourished patients (NRS-2002 > 3) compared the energy and protein intake of patients receiving two drinks/day of ONS (Nutridrink, Nutricia, Bulle, Switzerland; one drink equals 200 mL, 300 kcal, 12 g of protein) with (NT group) or without (ONS group) nutritional counselling from a dietitian during 10–15 days of hospital stay [22]. The NT group met the energy requirements before discharge by 107% and protein requirements by 94%, while the ONS group met the energy requirements before discharge by 90% and protein requirements by 88% [22]. Strotman et al. investigated, over two weeks, the impact of the implementation of several measures to improve the food service facilities (sensitisation of employees to the topic of food waste, train order assistance to optimise order taking, analysis of the flow of communication along the supply chain, configuration of a food catalogue with a detailed description of breakfast and dinner, change of order and delivery process, change of portion sizes according to target group-specific standards and their needs) on daily food waste per person and total food waste [31]. They found a significant decrease of 20% in the average quantity of food served daily per person in hospital (*p* < 0.0001). However, the average food waste rate remained constant before and after implementing measures (25.6 +/− 4.6% vs. 26.3 +/− 4.4%) [31].

#### 3.4.5. Plant-Based Proteins Meal (*n* = 1) 

One study [37] investigated the effect of adopting vegetarian meals for seven days compared with meat-containing meals in 447 hospitalised patients. Mean total food waste was significantly higher (+11%) in meat-containing meal consumers than vegetarian meal consumers (293 g/plate vs 259 g/plate; *p* = 0.05). Significant differences across food categories were observed in terms of food waste. Vegetables were the most wasted category in meat-containing meals, while grains and vegetables were the most wasted category in vegetarian meals consumers.

## 4. Discussion

In this systematic review, we identified studies assessing which hospital food strategy could reduce plate waste and improve patient dietary intake. Several strategies have been studied, such as patients’ meal condition, food and menu (including ordering and menu choices), service system (including ordering electronic systems), and developing and training hospital staff and food provider staff). The outcomes gathered demonstrate that the improvement of food intake and reduced food waste are strongly associated. In hospitalised patients, increased oral food intake primarily influences the improvement of nutritional status, which is strongly related to many outcomes such as complications, length of hospital stay, survival outcomes, and hospital costs. Food waste reduction also represents an important topic since hospitals collaborate with the sustainability of the whole healthcare system and environment by lowering the impact from food production to the final landfilling of uneaten food. 

One of the studied food hospital services is the catering system *Steamplicity*—consisting of using raw, semi- or fully cooked chilled foods, plated in a central production unit, and delivered to the hospital ward into a sealed plate which is chilled and then heated in a microwave [21]. Two studies have compared this concept with traditional cook-chill food [20,21]. The advantages of this system are the quick preparation and service times, the menu choice with flexible serving times, and cooked food that is served immediately in a small preparation area. However, the results were controversial regarding an increase in energy intake and a reduction in plate waste. This could be explained by the fact that there is a reduced variability in portion size and meal components for patients. Moreover, a certain complexity in staff management is necessary, highlighting the need for more training and education for catering hospital staff.

Most studies compared an innovative room service system with a traditional food service model, and showed a significant increase in food intake and plate waste reduction in patients receiving room service [28,33,35]. Thus, tailoring the food choice and individualising the patients’ desire for food could be successful. Indeed, the rotating 14-day cycle menu adds to production waste and inefficiency by preventing the ability to use remaining prepared food post-meal service due to the fact that different dishes are required for the next meal period. On the contrary, room service offers a greater variety of menu items and the flexibility to choose the higher energy and protein products on demand, leading to improved nutritional intake. Interestingly, these results are confirmed in an inpatient paediatric setting, not included in the qualitative synthesis [38]. This Canadian prospective cross-sectional study assessed the effect of a room service model on satisfaction, food costs/waste, and macronutrient intake in paediatric patients. With room service, satisfaction significantly increased, food costs decreased at breakfast and lunch, and reductions in waste occurred at all meals, especially with an increase in energy, protein, carbohydrate, and fat intake during lunch [38]. Other studies compared an electronic bedside spoken meal ordering system with a traditional paper menu [26,32,34], finding a significant increase in energy and protein intake with BMOS compared with the paper menu. This system allows instant access to personalised meal orders, responding to the fluctuations of appetite and the need for smaller, more frequent meals in hospitalised patients, especially cancer patients [32]. Indeed, poor appetite is one of the factors influencing dietary intake, which relates to the patients’ mood and anxiety during hospitalisation [39].

Interestingly, although this electronic system is potentially more challenging to manage for older patients, the ability to make food choices is equivalent to a traditional paper menu [32]. All these positive results are linked with the personalisation of the food and the presence of support to meals; such conditions seem crucial to fulfilling energy/protein requirements and require human professional intervention. Regarding food waste, the effect of the technological ordering system remains to be discussed. Patients probably order more nutritionally dense items despite wasting similar amounts of food. Personalising meals through room service and electronic bedside food systems might improve food intake and reduce food waste. Hospital services may decrease the need for costly fortified supplements, as patients can obtain a more significant proportion of their energy and protein requirements from available menu items at a time of their choosing. The room service model demonstrated a reduction in food costs linked to reduced production waste and from patient trays (plate waste) [35]. Every hospital—including public hospitals—should assess, in its proper setting, whether room service reduces food waste and improves patient and hospital outcomes. 

The PM implementation—consisting of feeding assistance and minimising unnecessary interruptions (including ward rounds and diagnostic procedures) during mealtimes—has also been investigated [23,24,30]. Only a few positive improvements to nutritional intake have been identified regarding energy and protein intake. On the other hand, the texture and meal presentation improvements may be promising. In hospitalised patients, the visual appeal, as well as the texture of types of food, is part of the acceptance of diet. Recent results showed that consistency modification positively impacts food waste [29,36]. Despite the limited number of studies and sample size of the included studies [27,29], improving and adjusting the texture of meals appears to reduce food waste and increase, at the same time, patient satisfaction level. Thus, the effort from the food service provider and the hospital staff to improve the appearance and propose an extensive range of food texture variety would significantly counter plate waste.

Interestingly, a study which investigated only the presentation without changing texture, conserving the same ingredients and budget, was carried out [27]. The authors found that the improvement of meal presentation positively influenced the patients’ visual perception and even the taste/smell of the plate, leading to a substantial reduction in food waste [27]. Although not included in this systematic review, a recent study explored the impact of hospital food and beverage presentation in terms of packaging on dietary intakes of healthy older people in an Australian university-simulated hospital ward [39]. Each participant experienced a ‘sealed’ and ‘pre-opened’ meal and snack condition. This study showed that older people may find some packaged products (cheese portions, biscuit portions, water bottles, fruit cups, and milk bottles) the most difficult packs to open in both hospital and community settings. These emerging findings remain to be confirmed, but already demonstrate the need to adjust the plate presentation and the plate texture of each patient by trained dietitians during the hospital stay. Moreover, the time taken and number of attempts to open snacks or breakfast products should be evaluated in terms of ‘openability’ [39]. 

One included study investigated the effect of some educational procedures on food intake and food waste (i.e., the sensitisation of employees to food waste, train order assistance to optimise order taking, analysis of the flow of communication along the supply chain, change of order and delivery process, change of portion sizes according to target group-specific standards and their needs on food waste) [31]. The authors found a significant decrease in the quantity of food served daily [31]. These results confirm that the compliance of meal quality and quantity, the individualisation of food supply, and plate waste personalisation substantially reduce plate waste. Moreover, they highlight that efficient communication between all actors (food service provider, dietitians, and medical staff) is fundamental. 

This review also shows that clinical professionals such as dietitians may be involved not only in terms of improvements in texture of meals, but also in terms of compliance with ONS intake, especially in malnourished patients [22]. Patients receiving an efficient, individualised nutritional intervention with nutritional counseling increase the consumption of ONS and consequently energy and protein intake, compared to those without nutritional counseling only receiving ONS. Quality of life has also been assessed and a substantial improvement has been shown. Although the studied approaches are different and the results need to be confirmed, dietary measures and dietary counseling by trained nutrition specialists seem to be the key to improving energy and protein intakes, increasing satisfaction level and quality of life, and at the same time reduce food waste and consequently hospital costs.

Several limitations cannot be neglected. Food waste and energy/protein intake measurements represent the main limitations for all studies focusing on these outcomes. Indeed, they have been measured either by visual estimation or weight. The visual measurements are often based on subjective methods of monitoring patient food intake, such as plate diagram food estimation. Weight measure may be considered a more accurate measure of intake and waste and has been referred to as the imperfect gold standard optimising accuracy. However, both methods of measurements did not include waste from food preparation or food preparation surplus. Another limitation of this review is the heterogeneity of food strategies, which can be explained by the limited number of original studies on this topic. Such a small amount of evidence could be explained by a lack of consciousness by physicians and researchers on the importance of hospital nutritional support and meal services during hospitalisation. Moreover, when a malnutrition state is ascertained, efforts are devoted to prescribing ONS or using artificial nutrition, rather improving hospital meals. 

## 5. Conclusions

This review demonstrates that monitoring and improving hospital services could improve food intake and reduce food waste. Applying changes in the service system, menus, serving time, patients’ needs, training staff, communications, quality of food, and meal conditions can lead to increased compliance with patients’ meals and a reduction in food waste. Although further studies should confirm the results to ascertain the full benefits and costs of these systems, the personalisation of the meal in terms of texture, presentation, and efficient room service appears to be a combination of efficient hospital strategies to improve the nutritional intake of patients and reduce food waste. Beyond any potential hospital food services studied, this paper also suggests that clinical nutritionist staff—especially dietitians—have a crucial role in achieving the reduction in plate waste and increasing patients’ food intake through the daily and active monitoring of the nutritional status and nutritional needs of hospitalised patients. 

## Figures and Tables

**Table 1:** Inclusion criteria according to PICOS criteria.

Criteria	Definition
Participants	Hospitalised patients ≥ 18 years old
Intervention	Any hospital food delivering practices
Comparator	Any comparator
Outcomes	Plate waste (in % or in kg), total food waste, food intake (%), energy intake (% or in kcal), protein intake (% or in g)
Study design	Peer-reviewed original experimental studies

**Table 2:** Characteristics and outcomes of included studies (classified by chronological order).

First Author, Year of Publication, Country	Study Design	Sample Size	Patient Type	Setting	Intervention Type	Time of Observation	Endpoints	Methods of Assessment	Results
Hartwell, 2003 UK [19]	Observational	*n* = 62	Women’s Health (*n*= 42) and orthopaedic (*n* = 20) inpatients	Hospital wards (*n* = 2)	Bulk trolley system = patients choosing food and amount from the trolley vs. Plated meal system = meals are ordered in advance (24 h before consumption)	3 consecutive days before and after 6 months.	Nutrient intake Food waste	Food weighed pre- and post-consumption Microdiet computer software for nutritional content	No differences between the nutrient content of the food intakes (both lower than recommended dietary values) ↓ plate waste with the bulk trolley service (5.9% vs. 11.6%) but high waste left on the trolley (20.5%) with bulk trolley system.
Edwards, 2006 UK [20]	Observational	*n* = 52	Patients presenting a mixture of clinical conditions	NHS teaching Hospital	Steamplicity vs. Cook-chill food service	3 consecutive days in 2-weeks periods for each arm	Food intake Food waste	Food weighed before and after the meal using digital weighing scales (individual food components separated when possible)	↑ food intake in Steamplicity than cook-chill system (daily mean of 282 g vs. 202 g at lunch; 310 g vs. 226 g at dinner) ↓ food waste in Steamplicity system then in cook-chill service (33% vs. 49%)
Hickson, 2007 UK [21]	Observational	*n* = 57	Patients presenting a mixture of clinical conditions; not at nutritional risk, without eating problems; and able to choose a menu	Hospital wards (*n*=7)	Steamplicity vs. Traditional bulk cook-chill system	1 lunch meal/patient between March and April 2006	Energy and protein requirements met with Steamplicity Energy and protein consumption between the two systems	Estimates of served food portion sizes Food waste weighed to calculate food intake and energy and protein intake (Nutritional analysis program) Comparison consumption and patient requirements	Steamplicity does not meet the patients’ energy requirements (36% deficit) ↓ energy intake in Steamplicity than in bulk cook-chill system (*p* = 0.04) No difference in protein intake between the two systems No difference in food wasted; more protein wasted in the Steamplicity system
Rufenacht, 2010 Germany [22]	RCT	*n* = 36	Hospitalised patients with NRS-2002>3	Internal Medicine hospital ward	NT: Nutritional counselling with a dietitian + ONS vs. ONS (without nutritional counselling)	10–15 days	Energy intake Protein intake	Weighing of all meals before and after consumption Energy and protein intake calculated with nutritional software	NT group met the energy requirements by 107% and protein requirements by 94% ONS group met the energy requirements before discharge by 90% and protein requirements by 88%
Hickson, 2011 UK [23]	Observational	*n* = 253	Hospitalised adult patients at high risk of malnutrition	Two large teaching hospitals	“Protected mealtimes” (PM) vs. Standard food service	June/July 2008: standard food service October/November 2009: PM	Nutritional (energy and protein) intake Food waste	Direct observation of meal consumption Weighing food consumed and food waste Evaluation of the intake by nutritional software	No impact of PM on energy intake (*p* = 0.25) ↓ protein intake (*p* = 0.04) in intervention group
Manning, 2012 Australia [24]	Monocentric observational	*n* = 23	Elderly inpatients (almost all at risk of malnutrition)	Hospital 2 wards	Volunteer feeding assistance program vs. No volunteers (feeding provided by nurses)	2 days for each arm	Energy and protein intake and % of energy and protein requirements met Food waste	Weighing of remaining food after meal consumption % of each item consumed Estimated energy and protein intake according to requirements	↑ energy and protein intake at lunch (*p* = 0.005; *p* = 0.009) No difference in daily total energy intake (*p* = 0.113) ↑ total daily protein intake (*p* = 0.004) ↑ % of energy requirements met with volunteers (64% vs. 58%, with an additional 448 kJ) ↑ % of estimated protein requirements met with volunteers (71% vs. 59%, *p* = 0.003).
Young, 2012 Australia [25]	Prospective pre-post	*n* = 254	Inpatients aged >65 years	Internal Medicine wards (*n* = 3) of a large metropilitan Hospital	3 mealtime assistance interventions: PM AIN: A nutritional focused staff-member assisting patients with meals PM + AIN: combined intervention	1 day in the first week of hospitalisation	Daily energy and protein intake	Visual estimation of plate waste (none, 1/8, 1/4, 1/2, 3/4, all) Intake evaluation by nutrient analysis software.	↑ energy intake, no differences between intervention groups (*p* = 0.16) ↑ protein intake (*p* = 0.07), no differences between the three interventions (*p* = 0.20). ↑ adequate EER (*p* < 0.01), no difference between interventions (*p* = 0.29). ↑ adequate protein intake (intake > EPR) (*p* = 0.03); no difference between interventions (*p* = 0.57).
Maunder, 2015 Australia [26]	Prospective	*n* = 119	Hospitalised adult patients	Private hospital	Bedside electronic meal ordering system (BMOS) vs. Paper menu (PM) group with default meals	48 h period × 2	Energy intake Protein intake	Use of photography and five-point visual wastage scale (0%, 25%, 50%, 75% and 100% wasted). Estimation of dietary intake by total meal eaten weight and calculated by nutritional analysis software analysis	In BMOS vs. PM group: ↑ energy intake: 8273 vs. 6273 kJ/day (*p* < 0.05) ↑ protein intake: 83 vs. 66 g/day (*p* < 0.05)
Navarro, 2016 Israel [27]	Prospective	*n* = 206	Adult hospitalised patients	Hospital Internal medicine ward	Improved meal presentation vs. standard lunch	Mean 4.7 days (intervention group) Mean 5.25 days (control group)	Food intake Food waste	Digital Imaging Method and visual estimation of plate waste (6-point scale: 0%, 25%, 50%, 75%, 90%, 100%) Estimation of food intake by nutritionDay questionnaire	+19% of food intake in the intervention group compared with control group (*p* < 0.05) ↓ starch and main course waste in the intervention group compared with control group (*p* < 0.05)
Collins, 2016 Australia [28]	Parallel controlled pilot study	*n* = 124	Elderly subacute patients (38% malnourished at admission)	Hospital, subacute geriatric ward	Modified hospital menu with higher energy foods including ONS (and a visual menu) vs. Control group: standard cook-chill meals (no visual menu)	14 days/group	Nutritional (energy and protein) intake Food waste	Visual estimation of plate waste before and after meal consumption Calibrated seated scales or self-reported or medical notes (if unable to be measured) Daily energy (kJ) and protein (g) intake estimated from plate waste data by nutritional software	In intervention vs. control group: ↑ mean energy intake (132 vs. 105 kj/kg/day; *p* = 0.003) ↑ mean protein intake (1.4 vs. 1.1 g protein/kg/day; *p* = 0.035)
Farrer, 2016 Australia [29]	Prospective	*n* = 65	Acute care inpatients prescribed smooth pureed meals	Acute care hospital	Smooth pureed meals in a moulded format (intervention group) vs. Smooth pureed meals in the standard format (control)	2 weeks	Food intake Plate waste	Weighing meal wastage with calibrated electronic scales	↑ food intake from <1/4 to >3/4 of the meal in the moulded form (*p* = 0.03) compared with control ↓ 120 g of plate waste in the intervention group compared with control group even if not significant (*p* = 0.09)
Porter, 2017 Australia [30]	RCT	*n* = 149	Admitted to the subacute setting	3 hospitals 3 wards n.2 geriatric evaluation and management wards and n.1 rehabilitation wards	PM (Intervention period) vs. Usual care (Control period)	4 weeks	Daily energy intake Daily protein intake Daily energy deficit	One quarter portion method per day; per patient per meal period and per interruption Use of nutritional software to estimate energy and protein intake	No significant differences between the intervention and control conditions for unadjusted analysis. ↓ energy deficit in intervention periods vs control periods if adjusted for age, nutritional status and type of subacute ward.
Strotmann, 2017 Germany [31]	Case study	*n* = 367	Hospitalised patients	Hospital surgery	A package of measures including: - Sensitisation of employees to food waste - Order assistance training - Analysis of the flow of communication along the supply chain - Configuration of a food catalogue with detailed description of meals - Change of order and delivery process - Change of portion sizes according to target group-specific standards and their needs vs. Usual care	2 weeks	Daily food waste rate (per person) Total food waste rate	Weighing food before and after consumption using electronic scales	↓ 20% in the average quantity of food served daily per person in hospital (*p* < 0.0001) No difference in hospital total waste; rate remained the same after implementing measure
Barrington, 2018 Australia [32]	Observational prospective	*n* = 96 (control) *n* = 105 (intervention)	Oncologic hospitalised patients	Hospital	BMOS vs. PM group with default meals	2 weeks	Total food intake Energy intake Protein intake Food waste	Use of photography and five-point visual wastage scale (0%, 25%, 50%, 75% and 100% wasted). Estimation of dietary intake by total meal eaten weight and calculated by nutritional analysis software analysis	↑ average energy intake (*p* < 0.001) in BMOS ↑ average protein intake (*p* < 0.001) in BMOS ↑ in receiving the food ordered (*p* < 0.001) in BMOS ↑ in choosing food that patients liked (*p* = 0.006) in BMOS No significant differences in average plate waste between the groups (34.3% in the BMOS vs. 35.3% in PM, *p* = 0.75)
McCray, 2018° Australia [33]	Retrospective analysis of data pre- and post-intervention	*n* = 148	Case mix of patients (general medical, surgical, and oncology wards)	2 adult care hospitals	Room service (RS) = meals ordered by patients from a “a la carte menu” and delivered within 45 min vs. Traditional foodservice model = meals ordered completing a paper menu (cook fresh, 14-day cycle) up to 24 h before meals	A 24-h consecutive period for 4 days	Nutritional intake Energy and protein intake as % of requirements Food waste	Meal intake observation tool using a five-point visual scale (0, 1/4, 1/2, 3/4, all) Nutrition analysis by nutritional software	In room service intervention vs. traditional foodservice model ↑ mean energy intake (1588 kcal/d vs. 1306 kcal/d; *p* < 0.005) ↑ mean protein intake (65.9 g/d vs. 52.3 g/d; *p* < 0.003) ↑ % of requirements of energy (75.1 vs. 63; *p* < 0.024) and protein (84.7 vs. 65; *p* < 0.011) intake ↓ total mean plate waste (12% vs. 29%; *p* < 0.001)
Mc Cray, 2018b Australia [34]	Prospective	*n* = 187	Adult hospitalised patients	Acute care hospital	Food and Nutrition Solutions (FNS) and Room Service Choice^TM^ vs. Traditional model (TM) with paper menu	4 days	Energy intake Protein intake Plate waste	Meal intake observation tool using a five-point visual scale (0, 1/4, 1/2, 3/4, all) Calculation of the nutritional intake using the FNS software	Compared with TM group, in FNS group: ↑ energy intake: 6379 vs. 5513 kJ/day (*p* = 0.020) ↑ protein intake: 74 vs. 53 g/day (*p* < 0.001) ↑ % of energy requirements met: 78% vs. 64% (*p* = 0.002) ↑ % of protein requirements met: 99% vs. 70% (*p* < 0.001) ↓ total average plate waste 17% vs. 30% (*p* < 0.001)
Neaves, 2021 Australia [35]	Retrospective analysis	*n* = 210	Adult hospitalised patients	Large tertiary hospital 3 wards: surgical, thoracic, cystic fibrosis	RS vs. Thaw-retherm service control group	5 weekdays	Nutritional (energy and protein) intake % of energy and protein met Food waste	Visual tool for nutritional intake and plate waste five-point visual scale (0%, 25%, 50%, 75%, 100%) and weight estimation of % wasted food	In RS compared to control group ↑ average energy and protein intake (*p* < 0.001). ↓ plate waste (15% vs. 40%) and production waste (5.6% vs. 15%, *p* < 0.001) ↓ food waste (*p* < 0.01) ↓ total average production waste (*p* < 0.001)
Razalli, 2021 Malaysia [36]	Cross-sectional	*n* = 95	Adult patients prescribed with texture-modified diet	Hospital	Texture modified diets 3 types: -Blended diet -Mixed porridge -Minced diet	from 1 to over 7 days	% plate waste % protein plate waste	Visual estimation of plate waste through Visual Comstock Scale (6-point scale: 0%, 25%, 50%, 75%, 90%, 100%) Digital food weighing scale	↑ plate waste (65%) in blended diet (65%) than minced diet (56%) and mixed porridge (35%) (based on weighing method) ↑ protein waste (61.1%) in minced diet compared with other diets (based on weighing method)
Berardy, 2022 USA [37]	Prospective	*n* = 447	Adult hospitalised patients	Hospital	Type of protein source vegetarian meals (peanut butter, tofu, black beans, brown lentils and hummus) vs. meat-containing meals	7 days	Total food waste Food waste of categories of food	Weighing of containers removing container weight Use of recipes for composite foods to determine proportional weights for individual categories of food	↑ 34.05 g of food waste (*p* = 0.05) in patients with meat-containing meals compared with vegetarian meals Largest category of food waste in meat-containing meals: vegetables. Largest category of food waste in vegetarian meals: grains and vegetables

Abbreviations: AIN, Additional assistant-in-nursing; BMOS, bedside electronic meal ordering system; EER, estimated energy requirements; EPR, estimated protein requirements; FNS, Food and Nutrition Solutions; NRS-2002, nutritional risk score—2002; NT, nutritional therapy; ONS, oral nutritional supplement; PM, protected mealtimes; REE, resting energy expenditure; RCT, randomised controlled trial; RS, room service; TM, Traditional Model; UK, United Kingdom; vs., versus; ↑ increase; ↓ decrease.

## Data Availability

Not applicable.

## Supplementary Materials

The following supporting information can be downloaded at: https://www.mdpi.com/article/10.3390/nu15020310/s1, Table S1: Full search strategies for electronic databases; Table S2: Quality assessment of the retrospective studies according to the Newcastle-Ottawa Scale; Table S3: Quality assessment of two randomised controlled trials according to the Cochrane risk of bias tool from *Cochrane Handbook for Systematic Reviews of Interventions*.
