# Supplementary material for: Hospital Services to Improve Nutritional Intake and Reduce Food Waste: A Systematic Review
Source: Nutrients. 2023 Jan 7;15(2):310. doi: 10.3390/nu15020310 (PMC9864175; doi:10.3390/nu15020310)
Supplement: Supplementary file 1 [file nutrients-15-00310-s001.zip › nutrients-2127724-supplementary.pdf]

Supplementary file

Table S1: Full search strategies for electronic databases

**Database**	**PUBMED (Medline)**	
**Date**	**29.06.2022**	
**Search strategy**		**Results**
**#1**	(HOSPITAL AND FOOD) OR (MEAL AND SERVICE) OR (HOSPITAL AND MEALS) OR (HOSPITAL AND CATERING)	146,086
**#2**	(FOOD AND WASTE) OR (PLATE AND WASTE) OR (PROTEIN AND INTAKE) OR (ENERGY AND INTAKE)	24,252
**#3**	#1 AND #2	862
**Database**	**WEB OF SCIENCE**	
**Date**	**29.06.2022**	
**Search strategy**		**Results**
**#1**	(HOSPITAL AND FOOD) OR (MEAL AND SERVICE) OR (HOSPITAL AND MEALS) OR (HOSPITAL AND CATERING)	17,880
**#2**	(FOOD AND WASTE) OR (PLATE AND WASTE) OR (PROTEIN AND INTAKE) OR (ENERGY AND INTAKE)	42,087
**#3**	#1 AND #2	847
**Database**	**SCOPUS**	
**Date**	**29.06.2022**	
**Search strategy**		**Results**
**#1**	(HOSPITAL AND FOOD) OR (MEAL AND SERVICE) OR (HOSPITAL AND MEALS) OR (HOSPITAL AND CATERING)	52,087
**#2**	(FOOD AND WASTE) OR (PLATE AND WASTE) OR (PROTEIN AND INTAKE) OR (ENERGY AND INTAKE)	45,170
**#3**	#1 AND #2	1324

Table S2. Quality assessment of the observational studies according to the Newcastle-Ottawa Scale

	**Selection**				**Comparability**	**Outcome**			
**First author, publication year**	**1) Representativeness of the exposed cohort**	**2) Selection of the non-exposed cohort**	**3) Ascertainment of exposure**	**4) Demonstration that outcome of interest was not present at start of study**	**5) Comparability of cohorts on the basis of the design or analysis controlled for confounders**	**6) Assessment of outcome**	**7) Was follow-up long enough for outcomes to occur?**	**8) Adequacy of follow-up of cohorts**	**9) Quality score**
Hartwell, 2003	*	*	*	*	**	*	*	*	**Good**
Edwards, 2006	Reduced sample size	*	*	*	**	*	*	*	**Good**
Hickson, 2007	Reduced sample size	*	*	*	**	*	*	*	**Good**
Hickson, 2011	*	*	*	*	**	*	*	*	**Good**
Manning, 2012	Reduced sample size	*	*	*	**	*	*		**Good**
Young, 2012	*	*	Pre - post study design	*	**	-estimation of the energy requirements of individual participants rather than measurement using indirect calorimetry -assessment of food intake on only one day of hospital admission	*	*	**Good**

	Selection				Comparability	Outcome			
Maunder, 2015	*	*	*	*	**	*	*	*	**Good**
Collins, 2016	Reduced sample size (pilot sutdy)	*	*	*	**	*	*	*	**Good**
Navarro, 2016	*	*	*	*	**	*	*	*	**Good**
Farrer, 2016	reduced sample size (high withdrawal rate due to patients not meeting the inclusion criteria)	*	*	*	* confounders to consider: age, gender, reasons for purea diet	responses mostly collected **verbally** from patients or their relatives by the investigator	*	high withdrawal rate due to patients not meeting the inclusion criteria	**Fair**
Strotmann, 2017	*	*	Pre - post study design	*	*	*	*	the reference and control measurement s took place in different seasons	**Good**
Barrington, 2018	*	*	*	*	**	*	*	*	**Good**
McCray, 2018a	*	*	*	*	* cohort different in terms of age, weight and medical classification	*	*	retrospective analysis of quality audit data over a 2.5-year period	**Good**

	Selection				Comparability	Outcome			
Mc Cray, 2018b	*	*	*	*	confounders to consider: age, medical classification	*	*	*	**Good**
Neaves, 2021	*	*	*	*	age statistically different between thaw-retherm and room service groups	*	*	*	**Good**
Razalli, 2021	*	*	*	*	**	*	*	*	**Good**
Berardy, 2022	*	*	*	*	*	no measurements of initial food weights	*	*	**Good**

Abbreviations: "*" means one star; "" means two stars; ND, not determined**

Good quality: 3 or 4 stars in the selection domain AND 1 or 2 stars in the comparability domain AND 2 or 3 stars in the outcome domain
Fair quality: 2 stars in the selection domain AND 1 or 2 stars in the comparability domain AND 2 or 3 stars in the outcome domain
Poor quality: 0 or 1 star in the selection domain OR 0 stars in the comparability domain OR 0 or 1 star in the outcome/exposure domain

Table S3. Quality assessment of two randomized controlled trials according to the Cochrane Risk of Bias tool from Cochrane Handbook for Systematic Reviews of Interventions

**First author, publication year**	**Random sequence generation**	**Allocation concealment**	**Blinding of participants and personnel**	**Blinding of outcome assessment**	**Incomplete outcome data addressed**	**Selective reporting**	**Other potential biases**
Rufenacht, 2010	**-**	**-**	+ due to the nature of intervention	**?**	**-**	**-**	**?**
Porter, 2017	**-**	**-**	+	**?**	**-**	**-**	**-**

-, low risk of bias; ?, unclear ; +, high risk of bias
